# Interaction of Sleep and Emotional Content on the Production of False Memories

**DOI:** 10.1371/journal.pone.0049353

**Published:** 2012-11-07

**Authors:** Shannon McKeon, Edward F. Pace-Schott, Rebecca M. C. Spencer

**Affiliations:** 1 Department of Psychology, University of Massachusetts, Amherst, Amherst, Massachusetts, United States of America; 2 Neuroscience and Behavior Program, University of Massachusetts, Amherst, Amherst, Massachusetts, United States of America; 3 Department of Psychiatry, Massachusetts General Hospital, Harvard Medical School, Boston, Massachusetts, United States of America; University of Granada, Spain

## Abstract

Sleep benefits veridical memories, resulting in superior recall relative to off-line intervals spent awake. Sleep also increases false memory recall in the Deese-Roediger-McDermott (DRM) paradigm. Given the suggestion that emotional veridical memories are prioritized for consolidation over sleep, here we examined whether emotion modulates sleep’s effect on false memory formation. Participants listened to semantically related word lists lacking a critical lure representing each list’s “gist.” Free recall was tested after 12 hours containing sleep or wake. The Sleep group recalled more studied words than the Wake group but only for emotionally neutral lists. False memories of both negative and neutral critical lures were greater following sleep relative to wake. Morning and Evening control groups (20-minute delay) did not differ ruling out circadian accounts for these differences. These results support the adaptive function of sleep in both promoting the consolidation of veridical declarative memories and in extracting unifying aspects from memory details.

## Introduction

During sleep, offline processing may help the brain reorganize and strengthen memories that have been encoded during the day [1]–[4]. Declarative memories are transferred from hippocampal to neocortical areas of the brain for long-term storage [1], [4]–[6] although the hippocampal formation may participate throughout the life of such memories [7], [8]. Notably neuroimaging studies also show that key components of the brain’s emotional memory circuit (which includes the amygdala as well as the hippocampus) activate more strongly during recall of previously learned emotional stimuli if sleep followed their initial encoding. [9], [10].

Memories are not an exact replica of experience but rather are constructed through the integration of multiple experiences and are therefore susceptible to distortion. The Deese-Roediger-McDermott (DRM) paradigm has been shown to elicit false memories in healthy individuals. In this paradigm, participants are presented with lists of semantically related words (“list words,” e.g., bed, rest, dream, awake). Not presented, however, is a “critical lure,” a word that semantically connects the words in each list (e.g., sleep). When asked to recall lists after a short break, participants consistently recall having studied critical lures, despite not having been exposed to them [11], [12].

A false memory can be created at one, some or all of memory’s sub-processes: encoding, consolidation, or retrieval. [13] Some researchers have divided the processes that cause the DRM effect into an activation/monitoring framework. [14] They suggest that a false memory can develop at encoding by one of two processes. The first is the associative-activation theory, which proposes that false recall is caused by spreading activation between existing conceptual representations, causing a word to be falsely remembered. The second is the “gist” (and the related “fuzzy trace”) theory, which suggests that subjects extract a gist in addition to the detailed memory of each word presented in the DRM lists. [15], [16] This gist representation summarizes the semantic feature that is common to the list words thereby activating the semantically related critical lure. [14] Accordingly, recall of a greater number of the critical lures may represent more efficient gist extraction during the encoding phase. It is also possible that a memory becomes distorted during the consolidation phase, particularly during sleep. During sleep, newly acquired memories are actively reorganized both to strengthen new memory traces and to become integrated with other memories in long-term storage.[13], [17]–[19] During this reorganization process, false memories can be created as a stable representation that did not occur originally but has been generalized to semantically associated knowledge. [13] False memories can also occur at the recall stage [20]. Within the activation/monitoring framework, this occurs when any monitoring- that is, memory editing or decision processes that aid in determining the origin of information- fails to suppress a false memory. [14] This effect has been found to occur more often under free recall procedures. [13] By giving subjects more monitoring tools, such as presenting each studied word with a pictorial representation, the DRM effect can be reduced. [14].

When DRM word lists are presented before sleep, subsequent recall for list words and false recall of critical lures both increase relative to recall following wake [21], [22] (but see [23]). Additionally, fMRI showed that, in individuals who slept normally but not those who were sleep-deprived on the first post-encoding night, the hippocampus and retrosplenial cortex were similarly activated for both list words and critical lures [14]. Therefore, over sleep, memory for the list words may become reorganized into a single coherent idea. These findings suggest that sleep may play a role in both preserving and generalizing memories.

In single-session experiments of emotional relative to neutral lists in the DRM paradigm, results are mixed. For example, Bauer et al. [24] found that emotion-word critical lures were falsely recalled at a higher rate than either concrete or abstract lures. This effect was carried primarily by positively valenced lures as opposed to negatively valenced ones. They also found that veridical recall was highest for concrete lists over abstract and emotional lists. A similar finding was reported by Dehon et al. [25]. However, Palmer and Dodson [26] found that, whereas veridical recall did not differ by emotionality, false recall of critical lures was highest for neutral word lists. Therefore, there is insufficient evidence to determine if, in a single session, the emotionality of studied words alters the degree to which false memories are generated in the DRM paradigm.

We examined how sleep modulates recall of emotional, relative to neutral, list words and critical lures. It has been demonstrated that sleep can preferentially consolidate emotionally negative over neutral material [27], [28]. One might therefore hypothesize that the preferential veridical recall of negative relative to neutral list words following sleep compared to wake would yield a similar pattern in false memory recall, i.e., greater negative false memory recall than neutral. However, preferential sleep dependent consolidation of emotional over neutral items has not been consistently found [29], [30]. Moreover, an alternative prediction is that memories that are preferentially protected might be the memories that are least susceptible to the distortion that yield false memories. Here we considered these alternatives using an emotional DRM paradigm in which veridical and false memory were probed following 12-hrs with overnight sleep or daytime waking.

## Methods

### Ethics Statement

This study was conducted in accordance with to the principles of Declaration of Helsinki, procedures were approved by the University of Massachusetts, Amherst Institutional Review Board and all participants provided written informed consent.

### Participants

Native English-speaking undergraduates at the University of Massachusetts, Amherst (mean age = 20 yr; SD = 0.97) participated for course credit. Participants had normal or corrected-to-normal vision, no history of sleep disorder, neurological disease, or head injury, were not taking sleep-affecting medications (excepting 3 taking SSRIs), and self-reported sleeping ≥6 h per night. Participants were asked to refrain from excess alcohol or caffeine on the day prior to the study and abstain from alcohol, daytime napping and excess caffeine during the study. Thirty participants self-selected into one of two experimental groups based on schedules and availability thereby becoming pseudo-randomly assigned to Wake (N = 15, 1 male) and Sleep (N = 15, 4 males) groups. Thirty-four participants similarly self-selected into one of two control groups (Morning: N = 17, 1 male; Evening: N = 17, 10 males).

### Test and Procedures

Ten 10-word DRM lists (5 lists containing only neutral words, 5 containing only negative words) were drawn from previous DRM studies [25], [31] and McEvoy’s normative associate database [32]. All lists were matched for backward associative strength (BAS), one of the best predictors of false recall using the DRM [31].

Lists were divided into 2 blocks of 5 lists counterbalanced across participants. In the Encoding phase, word lists were presented auditorially through headphones, one at a time, with a 3-sec within-list and a 20-sec between-list inter-stimulus interval. After Encoding, Sleep and Wake groups returned 12 hours later while Morning and Evening groups completed surveys for 20 min in the lab. In the subsequent Recall phase, participants were given 10 minutes to freely recall and type as many of the list words as they remembered and were fairly certain had been presented.

All participants completed the Stanford Sleepiness Scale (SSS [33]), Epworth Sleepiness Scale (ESS [34]), Morningness-Eveningness Questionnaire (MEQ [35]) and the Pittsburgh Sleep Quality Index (PSQI [36]). Sleep was self-reported using a retrospective diary for the night prior to the study day (Wake and control groups) or the night between sessions (Sleep group). Questionnaires were administered following presentation of the word lists during Session 1. Participants completed the SSS at each session.

### Analyses

Recall of list words was termed “Veridical Recall” and production of critical lures was termed “Lure Recall.” “Intrusions” were defined as falsely recalled words that were neither list words nor critical lures. Following Payne et al. [21], we defined “Adjusted Recall” as Veridical Recall minus the number of intrusions in order to adjust for participants’ guessing.

The effects of sleep and list emotionality were analyzed using 2-factor mixed Analyses of Variance (ANOVA) with one between-subjects factor, “Group” (Sleep vs. Wake or Morning vs. Evening) and one within-subjects factor, “Valence” (negative vs. neutral). The Greenhouse-Geisser correction was applied to within-subject main effects and interactions.

To control for heteroscedasticity and potential non-normality in distributions of Veridical, Adjusted and Lure Recall distributions, ANOVAs were repeated using a square-root transformation of data. For similar reasons, pairwise comparisons were repeated using the non-parametric Mann-Whitney U-test.

## Results

### Group Characteristics

Wake and Sleep groups did not differ in age, self-reported habitual sleep duration or PSQI score (unpaired t-tests). There was also no difference between the duration the Wake group slept on the night before their study day (mean 462 min, SD 53) and the duration the Sleep group slept (mean 470 min, SD 56) between sessions (p = .70). The Wake group showed trends toward greater ESS sleepiness, [*t*(29) = 1.80, *p* = .08] and MEQ morningness [*t*(29) = 1.89, *p = *.07]. The Sleep group showed significantly greater SSS sleepiness at Recall [*t*(29) = 3.34, *p* = .002].

### Veridical Recall

Participants in the Sleep group showed significantly greater Veridical Recall [main effect of Group: *F*(1,28) = 11.49, *p* = .002] and Adjusted Recall [*F*(1,28) = 10.01, *p* = .004]. Sleep group participants correctly recalled, on average, 6.5 more list words than the Wake group. The same ANOVA results were obtained using square-root transformed data.

**Figure 1:**
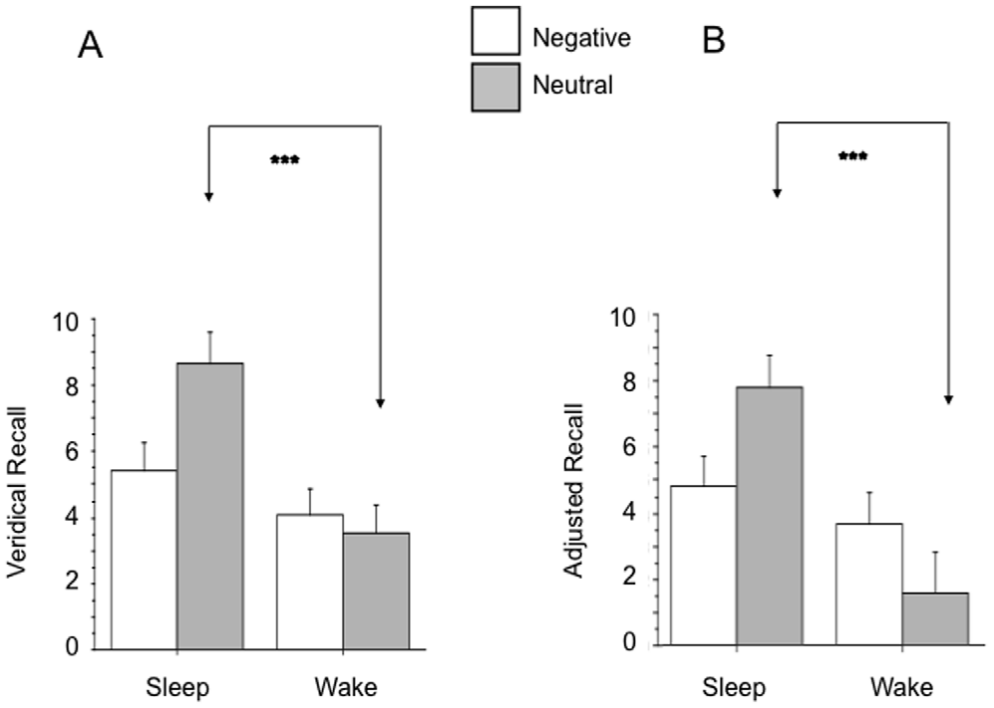
Comparison of Wake and Sleep groups for mean Veridical Recall of words in negative and neutral word lists (A) and mean Adjusted Recall (B). Error bars represent SEM. *** p<.001.

For Veridical Recall and Adjusted Recall, there were also significant Group × Valence interactions, [*F*(1,28) = 6.28, *p* = .018] and [*F*(1,28) = 8.95, *p* = .006] respectively. The Sleep group recalled significantly more neutral words than the Wake group for Veridical Recall [*F*(1,28) = 16.61, *p* = .0003] (Figure 1A) and Adjusted Recall [*F*(1,28) = 15.68, *p* = .0005] (Figure 1B). However, for negative words, there was no significant Group difference in either Veridical (p = .27) or Adjusted (p = .39) Recall (Figure 1). The same ANOVA results were obtained using square-root transformed data. Similarly, Mann-Whitney U-tests showed significant Group differences for neutral Veridical (U = 31.5, p = .0007) and Adjusted (U = 29, p = .0005) recall but not for negative Veridical or Adjusted Recall.

### Lure Recall

The Sleep group produced significantly more critical lures than the Wake group with a main effect of Group for Lure Recall [*F*(1,28) = 9.97, *p* = .004]. Participants in the Wake group recalled an average of 0.8 critical lures whereas those in the Sleep group recalled 2.27. The same results were obtained using square-root transformed data.

**Figure 2:**
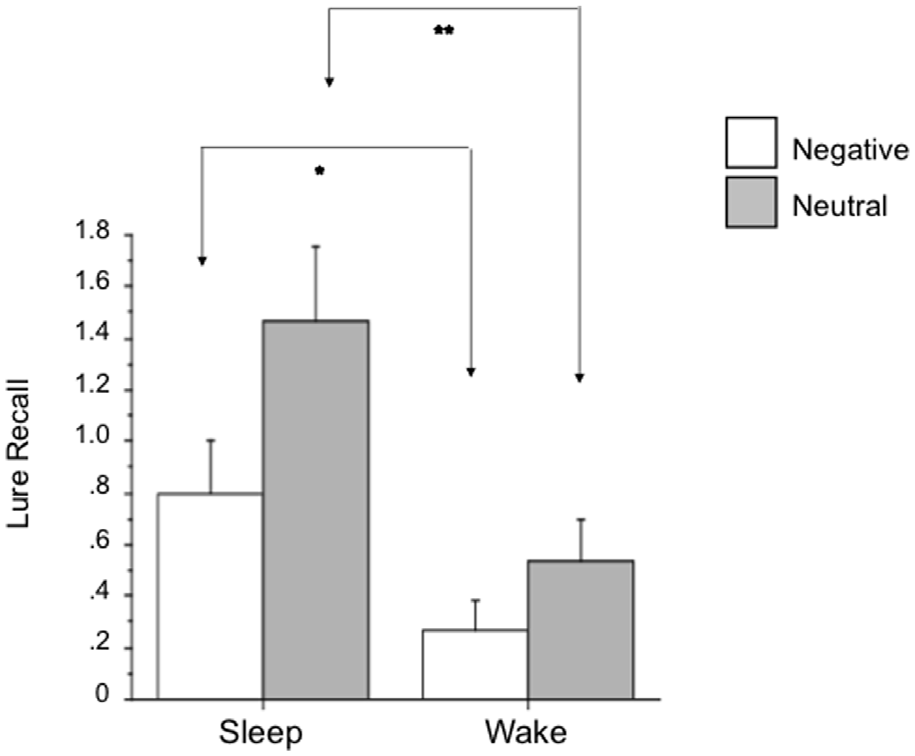
Comparison of Wake and Sleep groups for Mean Lure Recall of words in negative and neutral word lists. Error bars represent SEM. * p<.05, ** p<.01.

There was no Group × Valence interaction for Lure Recall (p = .25). Participants in the Sleep group had higher Lure Recall than the Wake group for both negative [*F*(1,28) = 5.27, *p* = .029] and neutral [*F*(1,28) = 7.80, *p* = .01] critical lures (Figure 2). Possible effects of group differences in guessing behavior were addressed by covarying each participant’s intrusions for each valence in its respective ANOVA. Including negative intrusions as a covariate for negative critical lures revealed a Group × covariate interaction [*F*(1,26) = 6.30, *p* = .019] precluding further comparison. However, including neutral intrusions as a covariate for neutral critical lures preserved the Group main effect [*F*(1,27) = 7.61, *p* = .01, Sleep greater]. The same ANOVA results were obtained using square-root transformed data. Mann-Whitney U-tests also showed significantly greater Lure Recall in the Sleep vs. Wake group for both negative (U = 69, p = .04) and neutral (U = 58.5, p = .02) lures.

### Intrusions

There was no significant difference in the number of negative (p = .56) or neutral (p = .32) intrusions between the Wake and Sleep groups. Similarly, there was no Group × Valence interaction (p = .21). These same results were obtained using square-root transformed data.

### Adjustments for Group Differences

Because there were more males in the Sleep group, analyses were repeated with females alone. Such analyses preserved the main effect of Group for Veridical [*F*(1,23) = 8.63, *p* = .007], Adjusted [*F*(1,23) = 6.37, *p* = .019] and Lure Recall [*F*(1,23) = 16.06, *p* = .0006]. Negative lists showed no Group difference for Veridical or Adjusted Recall but Lure Recall remained greater in the Sleep group [*F*(1,23) = 7.38, *p* = .012]. For neutral lists, Sleep-group females showed significantly greater Veridical [*F*(1,23) = 10.82, *p* = .003], Adjusted [*F*(1,23) = 9.56, *p* = .005] and Lure [*F*(1,23) = 12.94, *p* = .002] Recall.

To control for group differences in ESS, MEQ and Recall SSS, these three scores were added to analyses, individually, as covariates. Significant Group difference in neutral Veridical Recall was preserved adjusting for ESS [*F*(1,27) = 17.81, *p* = .0002], MEQ [*F*(1,27) = 11.64, *p* = .002] and Recall SSS [*F*(1,27)* = *13.74, *p* = .001]. Adjusting for ESS preserved significant group differences in Lure Recall for both negative [*F*(1,27) = 4.68, *p* = .04] and neutral [*F*(1,27) = 7.42, *p* = .011] lists. Adjusting for MEQ, the group difference remained a trend for negative [*F*(1,27) = 3.42, *p* = .076] and significant for neutral [*F*(1,27) = 7.39, *p* = .011] lists. Adjusting for Recall SSS showed a group difference trend for negative lists [*F*(1,27) = 3.95, *p* = .057] but a Group × SSS interaction [*F*(1,27) = 14.43, *p* = .0008] for neutral lists precluded further analysis.

### Control for Time of Day Effects

Indicative of absent time-of-day effects, there were no significant differences in Veridical Recall [F(1,32) = .11, p = .74], Adjusted Recall [F(1,32) = .39, p = .53], Lure Recall [F(1,32) = .10, p = .75] or Intrusions [F(1,32) = .11, p = .74] between Morning and Evening groups. This occurred whether negative and neutral word lists were examined together or separately and analyzing only female participants did not alter these results. The same ANOVA results were seen using square-root transformed data.

**Figure 3:**
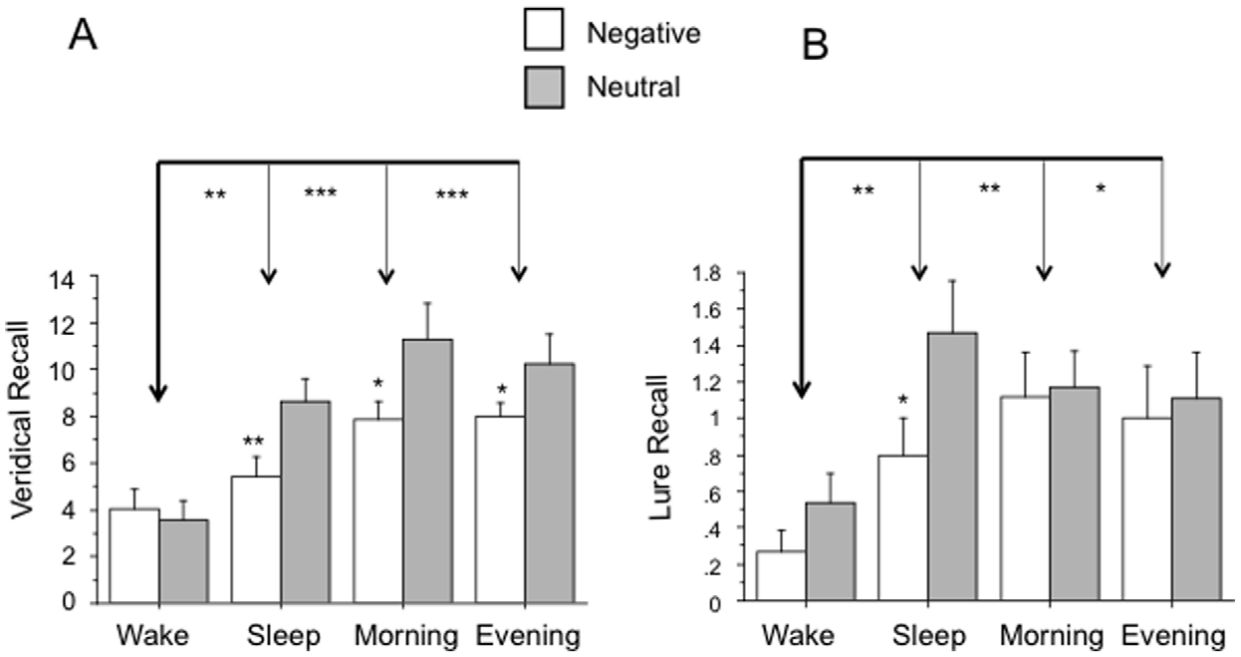
Comparison of Veridical Recall and Lure Recall across all participants including experimental (Wake, Sleep) and control (Morning and Evening) groups. Panel (A) shows Veridical Recall and (B) shows Lure Recall. Upper brackets depict post-hoc comparisons of the 4 groups collapsing across Valence. Asterisks directly above bars for negative items depict post-hoc comparison of negative vs. neutral items within individual groups for Veridical Recall (for which the Group x Valence interaction was a trend). * p<.05, ** p<.01, *** p<.001.

When considering all four groups with measures collapsed across Valence, there was a significant main effect of Group for Veridical [F(3,60) = 9.51, p = .0001], Adjusted [F(3,60) = 6.35, p = .0008], and Lure Recall [F(3,60) = 3.61, p = .018] but not Intrusions (p = .69). Post-hoc Bonferroni-Dunn tests showed that, for Veridical Recall (Figure 3A), Sleep (p = .009), Morning (p<.0001), and Evening (p<.0001) groups all significantly exceeded Wake, effects also seen for Adjusted Recall (p = .027 [trend], p = .0013, p = .0001 respectively). Similarly, for Lure Recall (Figure 3B), Wake was significantly exceeded by Sleep (p = .007), Morning (p = .006) and Evening (p = .014, trend). The same ANOVA results were seen using square-root transformed data.

Across all four groups, neutral items were preferentially recalled over negative ones (Valence main effect) for Veridical [F(1,60) = 14.38, p = .0003] (Fig 3A), Adjusted [F(1,60) = 4.75, p = .033] and Lure Recall [F(1,60) = 2.97, p = .09, trend] (Fig. 3B). Square root transformed data showed the same results for Veridical and Lure Recall but Adjusted Recall showed no Valence main effect. There was also a Group × Valence interaction for Veridical [F(3,60) = 2.49, p = .069, trend] and Adjusted [F(3,60) = 4.10, p = .01] Recall. Decomposing this interaction by Group for Veridical Recall shows that significantly more neutral words were veridically recalled in the Sleep, Morning and Evening groups relative to Wake (Figure 3A). Square root transformed data showed the same results except that the Evening group, like the Wake group, did not show a Valence main effect for Veridical Recall.

For Lure Recall, there were no interactions between Valence and Group (all 4 groups) (p = .36) or between Valence and the 2 delay durations (12 hr and 20 min) (p = .13). However, a Valence main effect appeared in the combined 12-hr delay (Sleep plus Wake) groups [F(1,28) = 7.54, p = .01] but not the 20-min delay (Morning plus Evening) groups (p = .90). More lures from neutral lists were recalled the Sleep group [F(1,14) = 5.39, p = .036] (Figure 3B) but this did not reach significance in the Wake group [F(1,14) = 2.15, p = .16]. Square root transformed data showed similar results but did not show a Valence main effect in either Sleep or Wake groups individually.

### Non-specific Memory and Floor Effects in Lure Recall

When Veridical Recall was added to the ANOVA model, Lure Recall remained a trend across the experimental groups [F(1.27) = 3.36, p = .078, Sleep greater] and a near-trend across all four groups [F(3,60) = 2.08, p = .11, absolute values of Morning > Sleep> Evening > Wake]. Because, recall was so low for negative, neutral and total lures across all 4 groups (0.815, 1.077 and 1.892 respectively) and across experimental groups alone (0.533, 1.0 and 1.533 respectively) the question arises as to whether floor effects in one or more groups limited the power of analyses. The percentage of individuals failing to recall any lures in the Wake group was greater than in Sleep, Morning and Evening groups (73% vs. 40%, 35% and 44% respectively). However, this difference failed to reach significance using Chi-Squared analyses for either all 4 groups together (p = .13) or across Sleep and Wake groups alone (p = .14).

## Discussion

These findings suggest that sleep plays a role in the formation of both negative and neutral false memories. Contrary to previous findings on visual recognition memory [27], [28], [37] and verbal memory [38], sleep increased Veridical Recall for neutral but not for negative list words. Moreover, sleep promoted Lure Recall for *both* negative and neutral word lists. Therefore, sleep and wake differentially affected Veridical Recall, but not Lure Recall based upon the emotionality of encoded content.

Addition of Veridical Recall to ANOVA analyses of Lure Recall decreased but did not eliminate the difference in Lure Recall between 12 h with sleep vs. continuous waking. This suggests that, although sleep enhancement of Lure Recall is proportional to it’s enhancement of general recall ability, it is not solely determined by a general sleep-dependent enhancement of memory. Similarly, because, like the 20-min delay control groups, approximately 2/3 of the Sleep group recalled at least 1 lure, the fact that only 1/3 of Wake-group individuals recalled at least one lure was not due to a floor effect resulting solely from difficulty in recalling any lures at all after a 12-hr delay.

Collapsing across valence categories, our findings are consistent with previous studies of sleep’s role in false memory formation [21], [22]. Payne et al. (2009) [21] found greater Lure Recall and Veridical Recall in the group that slept compared to the group that stayed awake between sessions. Diekelmann et al. (2010) [22] also found increased Lure Recall in the group that slept, but only if participants had low general memory performance. Like these studies, our findings contradict those of Fenn et al. (2009) [23] who found *decreased* Lure Recall following sleep compared to wake. However, Fenn et al. (2009) used a recognition test and, because free recall requires active retrieval of words, our participants may have relied more on their integrated memory of the word lists and thus spontaneously generated the critical lure more often following sleep.

In the current study, the sleep group recalled 23% of the 10 possible lures while the Wake group recalled only 8%. This rate of Lure Recall is lower than that reported Payne et al. [21] (Sleep 45%, Wake 36%). However, Payne et al. [21] examined only neutral words, and the ratio of lure to veridical neutral words for the Sleep and Wake groups in the current study (0.19 and 0.2 respectively) was almost identical to that of Payne et al. [21] (0.16 and 0.2 respectively). The chief difference between the two studies was, therefore, the lower overall rates of recall in the current study. This may be due to differences between Payne et al. [21] and the current study in numbers of lists (8 vs. 10 respectively), in numbers of words per list (12 vs. 10) or in the general frequency of occurrence or backward associative strength to critical lures of the specific words in lists. For example, relative to the current study, the larger number of words in the Payne and Diekelmann studies [11], [12] give more opportunities to form associations with the critical lure and, along with there being fewer lures to recall, may increase the percentage of such lures recalled. Notably, however, our results were similar to those reported by Diekelmann et al. (2010) [22] for their low-Veridical Recall performing subjects among whom significantly greater Lure Recall was seen following normal Sleep (36%) vs. normal Wake (12%). Diekelmann [22] did not observe sleep-dependent enhancement of Lure Recall in high-Veridical Recall performing subjects. Therefore, based upon this latter finding and because Veridical Recall in the current study was low in comparison to both Diekelmann et al. [22] and Payne et al. [21], participants in the current study, as low-Veridical Recall performing subjects, would be predicted to show sleep-dependent enhancement of Lure Recall.

Given that the Wake group showed lower Veridical, Adjusted and Lure Recall than both the Sleep and the two control, 20-min delay, groups, an alternative to the above postulated sleep-dependent enhancement of Lure Recall is that solely the effects of remaining awake over a 12 h period (e.g., interference effects) leads to both degraded Veridical and Lure Recall. However, comparison of total (negative plus neutral) Veridical Recall between the Sleep group and the combined control groups reveals significantly less Veridical Recall [F(1,47) = 5.02, p = .03] but not Lure Recall (p = .90) following the longer delay (see Figure 3). Therefore, although a longer delay contributes to loss of Veridical Memory, this effect is not paralleled by Lure Recall, suggesting that a sleep-dependent enhancement, or at least protection, of Lure Recall occurs when sleep intervenes across a longer delay.

Contrary to many studies that have shown enhancement of declarative memory by emotional content (e.g., [39], reviewed in [40], [41]), the main effect of Valence paradoxically favored Veridical Recall of neutral words (Figure 3A). One possible explanation, based on gist theory [15], [16], is that emotional context may enhance gist memory at the expense of specific details [42]. Examination of Figure 3A shows that, in both Evening and Morning groups, preferential Veridical Recall of neutral words was observed after only 20 minutes. This suggests that the specific, veridical items in the neutral vs. negative lists may have already achieved preferential status at encoding. Perhaps more of a limited neurocognitive resource is allocated to gist when items are emotionally charged. It is notable that the bias for neutral words, present for Veridical Recall after only 20 min, does not appear in Lure Recall until 12 h have elapsed and, even then, only significantly following sleep. Therefore, based upon theories of the DRM effect that focus on memory retrieval [20], both time and sleep may be required for the better-encoded neutral veridical details to be consolidated and incorporated into associative networks to then support the preferential extraction of neutral vs. negative gist at retrieval.

However, an alternative explanation of these results is that sleep may not preferentially enhance negative emotional word pairs. A number of recent studies [29], [30] have failed to find preferential enhancement of negative relative to neutral stimuli following sleep vs. wake. In one such study, Campanella and Hamann (2011) [43] found no difference in sleep dependent performance changes for neutral and negative word pairs, consistent with the present results. Rather, sleep is likely to give preference to memories that are salient and have future relevance (e.g., [44], [45]). Supporting this, a recent study has shown that neutral word pairs were better recalled after sleep than after wake only when participants were told of the need for later recall (i.e., intentional learning) and not in a condition in which the recall probe was a surprise (i.e., incidental learning) [39]. While participants in the present study knew of future recall, explaining the observed enhancement of recall in the Sleep relative to Wake group, emotional words like “gloomy” are likely of no more future relevance than “aroma” (see [46]). An additional possibility is that the verbal stimuli used in the current DRM paradigm may not evoke sufficient emotional response to produce the emotional memory effects seen with more powerful visual [27] or narrative [38] stimuli.

Greater subjective sleepiness at retrieval in the Sleep group could have increased false recall if less attention was paid to the task. However, because there was no difference in either Veridical or Lure Recall between the single-session Morning and Evening groups, it is unlikely that a simple time-of-day effect caused observed differences.

List valence, delay duration and sleep all influenced Veridical and Lure Recall and current results represent their combined effects. Nonetheless, each effect may be individually adaptive. For example, preferential encoding of gist at the expense of detail for emotionally charged stimuli ensures that the most useful information is made available for consolidation. Sleep would then enhance consolidation of both gist and detail. In addition, sleep may promote associative processes that allow further gist extraction from encoded detail during offline integrative processing, at retrieval or at both times.
